# Anxious-Withdrawal and Sleep Problems during Adolescence: The Moderating Role of Peer Difficulties

**DOI:** 10.3390/bs13090740

**Published:** 2023-09-05

**Authors:** Julie C. Bowker, Jessica N. Gurbacki, Chloe L. Richard, Kenneth H. Rubin

**Affiliations:** 1 Department of Psychology, University at Buffalo, The State University of New York, Buffalo, NY 14260-4110, USA; jngurbac@buffalo.edu (J.N.G.); cr237@buffalo.edu (C.L.R.); 2 Department of Human Development and Quantitative Methodology, University of Maryland, College Park, MD 20742, USA; krubin@umd.edu

**Keywords:** anxious-withdrawal, sleep, peer exclusion, peer victimization, adolescence

## Abstract

Anxious-withdrawal is a well-established individual risk factor for psychosocial difficulties during adolescence. It is unknown, however, whether it also places youth at increased risk for physical health problems, such as sleep difficulties. This study examines the concurrent and prospective associations between anxious-withdrawal and six types of sleep difficulties (i.e., sleeping too much, sleeping too little, talking/walking in sleep, being overtired, nightmares, and general trouble sleeping). We further evaluate whether these associations differ for adolescents who are high versus low in exclusion and victimization. The participants were 395 adolescents (*M*_age_ = 13.61 years; 35% ethnic minority) who completed peer nominations of anxious-withdrawal, exclusion, and victimization at Time 1 (T1). Their mothers completed reports of sleep difficulties at T1 and at Time 2 (T2). Path analyses revealed unique associations between anxious-withdrawal and several types of sleep difficulties (e.g., sleeping too much) at T1. Analyses also revealed a significant interaction effect between T1 anxious-withdrawal and exclusion/victimization such that anxious-withdrawal was prospectively associated with trouble sleeping only for those young adolescents who are highly excluded/victimized. Our findings are the first to link anxious-withdrawal to a physical health outcome in adolescence and point to the need for future research to not only examine anxious-withdrawal and physical health but also to include assessments of peer difficulties.

## 1. Introduction

While most youth desire to engage with their peers, some youth regularly withdraw behaviorally when in the company of their peers [1]. When this behavioral tendency is rooted in social anxieties and fears, it is referred to as anxious-withdrawal (closely related constructs include anxious-solitude, social withdrawal, shyness–sensitivity, e.g., [2]). Understanding anxious-withdrawal is important, because it is related to a broad range of significant psychological difficulties, including anxiety, depressive symptoms, and loneliness [3,4]. Anxious-withdrawal during childhood and adolescence is also associated with numerous peer problems, such as peer exclusion (i.e., being left out of group activities) and victimization (i.e., being subjected to repeated peer abuse), which in turn explain why many anxiously-withdrawn youth suffer psychologically [2,3].

While the negative psychological and peer concomitants of anxious-withdrawal are well-established, less is known about potential related physical health problems. In recent years, it has become clear that peer difficulties during adolescence can foster physical health problems, including numerous types of sleep difficulties (e.g., poor sleep quality [5,6,7]). In general, the majority of Americans do not get the recommended amount of daily sleep, and this is especially the case for adolescents (10–18 years) [8]. Sleep complications are significant at any age because they are related to other types of physical and psychological health difficulties (e.g., anxiety, loneliness, obesity [9,10,11]). However, during adolescence, sleep difficulties also interfere with important developmental tasks, including those in the academic domain (e.g., studying, academic performance), and thus can lead to cascading negative developmental consequences [12,13]).

Anxiously-withdrawn youth are shy and anxious; they also experience problematic peer relations [14]. These intra- and interpersonal difficulties may independently, or interactively, predict sleep difficulties, a novel hypothesis evaluated for the first time in the current investigation. Once we better understand the many different types of health difficulties associated with anxious-withdrawal, we can use this knowledge to develop strategies that help anxiously-withdrawn youth cope with their difficulties and learn to more often approach, rather than avoid, their social worlds.

### 1.1. Anxious-Withdrawal and Sleep

Most published studies on anxious-withdrawal (and related constructs) have not evaluated the occurrence or development of sleep difficulties. There are several relevant lines of inquiry, however, suggesting that anxious-withdrawal might be related to sleep difficulties. First, it is well-established that anxiety contributes to the development of sleep difficulties during adolescence. Of note, in the extant literature, sleep difficulties can be indexed in numerous ways, including sleeping too much, sleeping too little, talking/walking in sleep, nightmares, general trouble sleeping, sleep quality or sleep efficiency (or percent time in bed sleeping), insomnia, nighttime awakenings, and daytime sleepiness. Anxiety during adolescence has been linked to all of these indices of sleep difficulty (e.g., [5,11]). Although there is some variability across different informants, recent research indicates positive associations between both self- and parent-reports of anxiety and self- and parent-reports of sleep difficulties during adolescence [15].

When clinical samples are considered, sleep problems are found to be extremely common in youth with diagnosed anxiety disorders. For instance, approximately 88 percent of youth with anxiety disorders report at least one type of sleep difficulty, and more than half report greater than three different types of sleep difficulties [16]. In addition, clinically elevated levels of anxiety in early childhood have been found to predict insomnia in middle adulthood [17]. Anxiety is strongly linked with a variety of sleep disturbances due to the heightened physical arousal associated with anxiety as well as the rumination and biased information processing in which many anxious youth engage [7]. Of course, not all anxiously-withdrawn youth report clinically elevated levels of anxiety, but all do withdraw from their peers due to (at least small-to-moderate levels of) social anxieties and fears [18].

Second, there is growing evidence that loneliness, or dissatisfaction with and perceived inadequacy in social relationships, is associated with numerous types of sleep difficulties [19]. For instance, lonely adults report poorer sleep efficiency than do non-lonely adults, such that they experience more restless sleep and spend more time awake after sleep onset [20]. Loneliness is also associated with longer sleep latency, more nighttime awakenings, and lower perceived sleep quality during emerging adulthood [21]. Similarly, in young adults, loneliness has been robustly related to lower sleep quality [22]. Although less commonly studied with samples of adolescents, there is some indication that loneliness similarly interferes with sleep quality during adolescence [23]. Thus, at all ages, loneliness appears to “invade the nights” [20] (p. 364), likely because it promotes such negative social cognitions as hypervigilance to social threats, which keep the individual physically aroused and alert and thus unable to fall and stay asleep [19]. Although anxiously-withdrawn youth actively avoid their peers, they also very much desire to be with them, and as a result, report strong feelings of loneliness [3,24].

Third, there is some indication that social isolation leads to sleep difficulties. In this area of research, social isolation is typically indexed by self-reports of few social interactions, small network sizes, and the lack of social support [25,26]. In addition, these indices have been related positively to several types of sleep difficulties, including insomnia, longer sleep latency, and poor sleep quality [25,26,27]. For instance, in a sample of young adults, individuals who rated themselves as socially isolated reported poor sleep quality, long sleep latencies, and high levels of daytime dysfunction [28].

The evidence for links between social isolation and sleep difficulties is most robust in studies of adults. However, several recent studies have revealed associations between social isolation and sleep difficulties during adolescence [26,29]. At any age, social isolation may act as an interpersonal stressor that promotes rumination and interferes with sleep. It is also plausible that the lack of social stimulation interferes with the extent to which individuals are appropriately exhausted and ready for nighttime sleep. By definition, anxiously-withdrawn youth spend considerable time alone and on the periphery of the social scene and thus are usually also socially isolated. That said, there are many youth who are socially isolated who are not also anxiously-withdrawn (e.g., many aggressive youth).

We were not able to locate a single study in which anxious-withdrawal during adolescence was examined as it related to sleep difficulties. However, several studies have shown significant associations between shyness and sleep difficulties during adulthood (e.g., [30]). Conceptually, the constructs of shyness and anxious-withdrawal are related with the shared anxiety and fear of negative evaluation; the difference between these phenomena, however, is that not all shy individuals are socially withdrawn [1]. Nevertheless, the available evidence suggests that anxious-withdrawal may be a risk factor for sleep difficulties during adolescence. The present study is novel in the evaluation of this possibility with the consideration of six types of sleep difficulties: sleeping too much, sleeping too little, talking/walking in sleep, being overtired, nightmares, and general trouble sleeping.

### 1.2. The Role of Peer Difficulties

The present study also considers the possibility that peer difficulties, in the form of peer victimization and exclusion, may moderate the prospective associations between anxious-withdrawal and sleep difficulties. As noted previously, it is well-established that anxious-withdrawal is associated with and predictive of peer difficulties [31]. This is likely because anxious-withdrawn behaviors are judged to be non-normative and atypical, especially during childhood and adolescence when peer interaction and relationship involvement is expected and valued [32]. It has also been posited that anxiously-withdrawn youth are likely judged to be “easy targets” for peer victimization who are unlikely to retaliate and/or fight back [33].

To our knowledge, no past research has considered anxious-withdrawal and peer problems in relation to sleep difficulties, but peer problems alone have been linked concurrently and prospectively with sleep difficulties during adolescence [34,35,36]. Moreover, it has been found that peer victimization moderates the associations between loneliness and sleep difficulties, such that loneliness is most strongly associated with sleep difficulties for those who are highly victimized by peers [22]. In this study [22], it was suggested that peer difficulties are an interpersonal stressor that likely exacerbates the sleep difficulties associated with the individual/intrapersonal risk factors of loneliness.

Thus, in the present study, we considered not only whether anxious-withdrawal might represent an intrapersonal behavioral risk factor for sleep difficulties but also whether interpersonal difficulties with peers might enhance the risk for developing increased sleep difficulties. This suggestion seems plausible if both intra- and interpersonal difficulties conspire by increasing intrapersonal stress, rumination, and negatively biased cognitions. Furthermore, this suggested developmental process may be especially likely to occur during early adolescence (10–14 years) when getting along with peers increases in importance, rumination tendencies first begin to develop and become resistant to change, and sleep becomes increasingly disrupted [37].

### 1.3. The Present Study

In summary, the present study extends past research with its novel consideration of the concurrent and prospective associations between anxious-withdrawal and six types of sleep difficulties (i.e., sleeping too much, sleeping too little, talking/walking in sleep, being overtired, nightmares, and general trouble sleeping). Peer difficulties in the form of peer exclusion and victimization were also evaluated as a moderator of the prospective associations between anxious-withdrawal and sleep difficulties. Due to the dearth of research in this area, hypotheses were tentative and not specific to different types of sleep difficulties. Instead, it was generally expected that anxious-withdrawal would be related significantly to sleep difficulties (Hypothesis #1) and that anxious-withdrawal in combination with peer exclusion and victimization would predict increases in sleep difficulties over time (Hypothesis #2). To evaluate these hypotheses, we utilized a longitudinal sample of young adolescents and multi-method assessments (maternal-reports of sleep, peer nominations of anxious-withdrawal and peer exclusion and victimization).

## 2. Materials and Methods

### 2.1. Participants and Procedures

Participants were 395 (*M*_age_ = 13.61 years (*SD* = 0.54) at the start of the study; 139 boys, *M*_age_ = 13.67 years (*SD* = 0.52); 256 girls, *M*_age_ = 13.56 years (*SD* = 0.55)) young adolescents in the Greater Washington, DC metropolitan area selected from a larger longitudinal project on peer relationships (see [14,33]). These participants (and their mothers) completed measures in Grade 5 and/or Grade 6 and also when they were in Grade 8 (described in more detail below). The sample was racially diverse, with 35% belonging to a racial or ethnic minority group (with 11% African American, 17% Asian, and 5% Hispanic/Latino). In terms of parental education, 66% of the participants’ mothers (59% of the fathers) had an undergraduate or advanced graduate university degree, 19% had some college education (12% of the fathers), and 6% had high school or vocational education (12% of the fathers). Comparisons of participants who were included in this study versus those who were not included from the larger project did not reveal any significant differences in the study variables (output available by request).

In the larger project, principals of public elementary and middle schools were first contacted and agreed to participate in the study, and then all students in their schools were invited to participate. Youth with signed parental consent and adolescent assent forms then completed paper-and-pencil peer nomination measures in their schools during the spring of Grade 5 (the final year of elementary school) and/or Grade 6 (the first year of middle school; 70% consent rate; University of Maryland, Institutional Review Board #00475). All participating Grade 5 and 6 youth and their parents (392/395 or 99% of these parents were mothers) were next invited to complete additional measures in the laboratory (where they provided additional signed parent consent and adolescent assent).

The school and laboratory measures collected during Grade 5/Grade 6 are referred to as occurring at Time 1 (T1). Most (75% of the sample) of the T1 data were collected from participants and their mothers when the participants were in Grade 6. There were no significant grade (Grade 5 versus Grade 6) differences, however, in any of the T1 study variables (*p*s > 0.12). Also of note, when participants completed school and laboratory measures in both Grades 5 and 6, only their Grade 6 data were used. Participants and their parents also completed measures in the laboratory or at home when they were in Grade 8 (Time 2; T2; the final year of middle school). In addition to the measures described next, participants completed additional measures, such as a nomination measure of friendship at the T1 school visits and a social information processing measure at the T1 laboratory visits; these were not of interest in this investigation. Participants received gift cards for completing the laboratory measures at each time point.

### 2.2. Measures

#### 2.2.1. School Measures (T1)

**Anxious-withdrawal and peer exclusion/victimization.** Participants (and their classmates) completed a 30-item peer nomination measure that included four items descriptive of anxious-withdrawal (e.g., “very shy”, “gets nervous about participating in group discussions” [14,33]). Four items descriptive of peer victimization and exclusion were also included (e.g., “gets hit/kicked”, “left out of group activities”). In Grade 5, participants selected one same- and one other-sex peer from their classroom and grade who were most like these items from rosters; in Grade 6, due to school schedules that involved numerous classroom changes throughout the day, participants wrote names for up to three same- and other-sex grade-mates without a roster. Self-nominations were permitted but not considered. For each item, the nominations received were summed, proportionalized, and then standardized within sex and school (to adjust for differences across schools in the number of possible nominations received [38]). Mean scores were calculated with higher scores reflecting greater anxious-withdrawal (α = 0.84) and peer exclusion/victimization (α = 0.90). Previous psychometric work with the Grade 5 and Grade 6 peer nomination data revealed identical and separate anxious-withdrawal and peer exclusion/victimization factors in each grade [14].

#### 2.2.2. Laboratory Measures (T1, T2)

**Sleep problems**. Sleep problems at T1 and T2 were measured with the same six items drawn from the parent-reported Child Behavior Checklist (CBCL; [39]) (“nightmares”, “sleep less than most kids”, “sleep more than most kids”, “talks/walks in sleep”, “overtired”, “trouble sleeping”; for other published studies of 6-to-17-year-old youth that utilized the CBCL to assess sleep, see [15]). At both time points, items were scored on a three-point scale (ranging from 0 = not true to 2 = very/often true). Items were analyzed individually rather than with composite/mean scores due to poor internal consistencies at each time point (αs < 0.50). Self-reports of five of these sleep difficulties (“nightmares”, “sleep less than most kids”, “sleep more than most kids”, “overtired”, “trouble sleeping”; with the Youth Self-Report; YSR [40]) were also available, but at T2 only. Therefore, primary models utilized the CBCL data, but an exploratory model was also evaluated with the T2 YSR self-report data utilized.

### 2.3. Data Analysis

**Figure 1:**
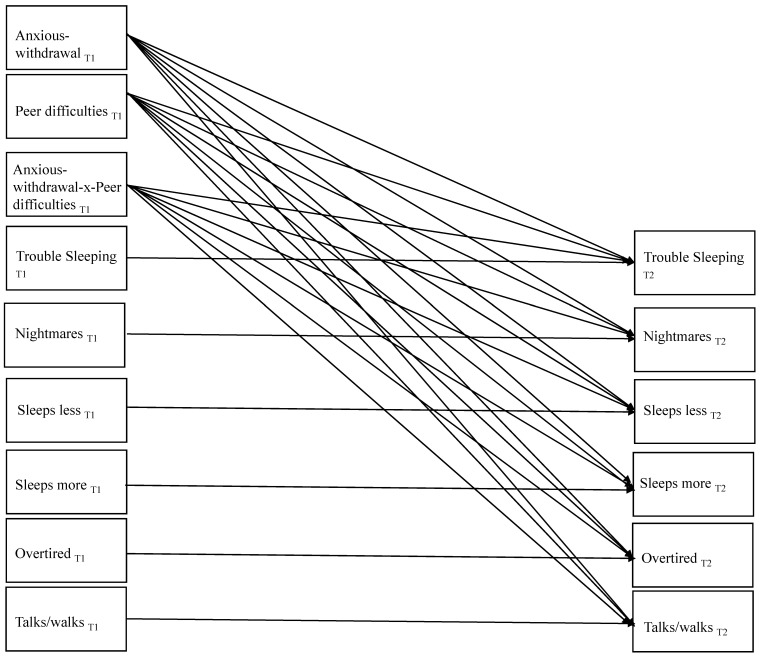
Model Evaluated in the Primary Analyses Predicting Time 2 Sleep Difficulties.

Means and standard deviations for, and zero-order correlations among, the study variables were first examined and are presented in Table 1. To evaluate the primary study objective and hypotheses, Mplus version 6.12 [41] was used to estimate a path model with full information maximum likelihood estimation with robust standard errors. Missing data were minimal, and full information maximum likelihood estimation is appropriate to handle missing data. The path model is depicted in Figure 1 with the main effect from anxious-withdrawal to sleep difficulties included as a test of Hypothesis #1. The stability paths (from T1 to T2) between the sleep variables were also estimated, as were the paths from the interaction term between the centered T1 anxious-withdrawal and exclusion/victimization variables and the T2 sleep problem variables (with the interaction effect included to test Hypothesis #2). Although not shown in the figure, covariances between exogenous variables (each T1 maternal-reported sleep problem variable, T1 anxious-withdrawal, T1 exclusion/victimization) were estimated, as were the covariances between the endogenous variables (each T2 maternal-reported sleep problem variable). Model fit was assessed with chi-square goodness-of-fit and the root-mean-square error of approximation (RMSEA; 0.08 or less), standardized root-mean-square residual (SRMR; 0.09 or less), and comparative fit index (CFI; 0.95 or greater). Only significant effects are described.

## 3. Results

### 3.1. Preliminary Analyses

In the correlational analyses, anxious-withdrawal at T1 was related positively to T1 exclusion/victimization, T1 maternal-reports of nightmares, T1 and T2 maternal-reports of sleeping more than other kids, and T1 and T2 maternal-reports of trouble sleeping. A series of exploratory *t*-tests did not reveal any significant sex differences in any of the study variables (*p*s > 0.05).

### 3.2. Primary Analyses

**Figure 2:**
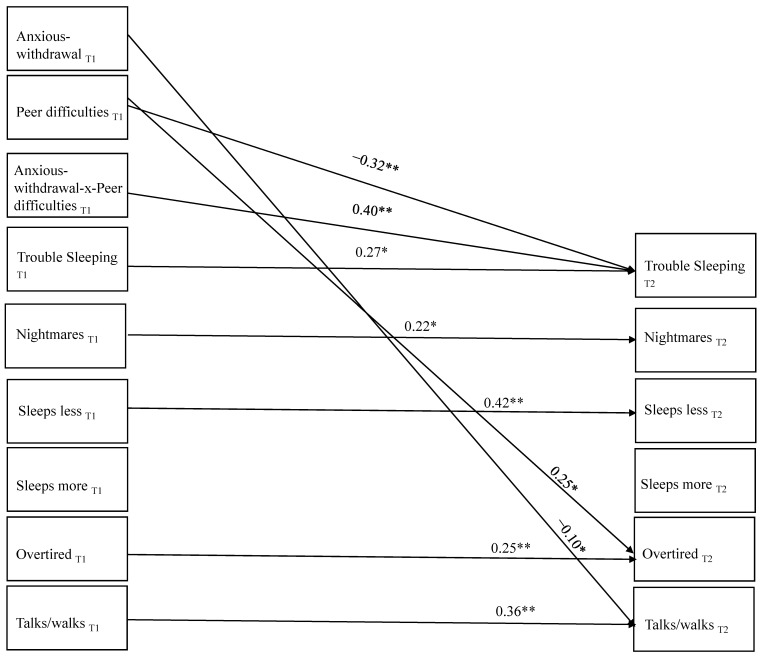
Significant Paths in the Primary Model Predicting Time 2 Sleep Difficulties; * *p* < 0.05, ** *p* < 0.001.

There was a good fit of the model to the data: χ^2^ (30) = 40.50, *p* = 0.096, RMSEA = 0.03, 90% CI [0.000, 0.051], SRMR = 0.048, CFI = 0.94; thus, no post hoc model fitting was performed. As is evident in Figure 2, significant stability was found over time for each type of sleep problem, with the exception of reports of sleeping more than other kids. In terms of additional prospective associations, T1 exclusion/victimization predicted increases in maternal-reports of being overtired and decreases in trouble sleeping over time. T1 anxious-withdrawal predicted decreases in maternal-reports of talking/walking in sleep from T1 to T2. In addition, the interaction between T1 anxious-withdrawal and T1 exclusion/victimization when predicting T2 maternal-reports of trouble sleeping was significant. Simple slope analyses showed that T1 anxious-withdrawal predicted increases in maternal-reports of trouble sleeping (β = 0.34, *p* = 0.001) at high levels of T1 exclusion/victimization but not at low levels (β = −0.15, *p* = 0.18).

Not shown in the figure for ease of communication were numerous unique within-time significant associations. For example, T1 anxious-withdrawal and T1 victimization/exclusion were significantly related (β = 0.31, *p* = 0.001). T1 anxious-withdrawal was also related uniquely to T1 maternal-reports of sleeping more than other kids (β = 0.16, *p* = 0.02) and trouble sleeping (β = 0.18, *p* = 0.01). T1 exclusion/victimization was associated uniquely with T1 maternal-reports of trouble sleeping (β = 0.16, *p* = 0.05) but was not related significantly with any other types of sleep difficulties at T1.

Within-time unique associations among the sleep difficulties also emerged. At T1, maternal-reports of nightmares were related uniquely to maternal reports of being overtired (β = 0.12, *p* = 0.04), sleeping less than other kids (β = 0.11, *p* = 0.05), sleeping more than other kids (β = 0.13, *p* = 0.04), talking/walking in sleep (β = 0.17, *p* = 0.007), and trouble sleeping (β = 0.29, *p* = 0.001). At T1, maternal-reports of sleeping less than other kids were also correlated with maternal-reports of being overtired (β = 0.26, *p* = 0.001), and maternal-reports of trouble sleeping were associated uniquely with maternal-reports of being overtired (β = 0.21, *p* = 0.004), sleeping less than other kids (β = 0.31, *p* = 0.004), and talking in sleep (β = 0.18, *p* = 0.03).

At T2, maternal-reports of nightmares were associated uniquely with maternal-reports of being overtired (β = 0.23, *p* = 0.044), talking/walking in sleep (β = 0.27, *p* = 0.01), and trouble sleeping (β = 0.31, *p* = 0.003). T2 maternal-reports of sleeping less than other kids were also related uniquely to maternal-reports of being overtired (β = 0.19, *p* = 0.037) and so were maternal-reports of sleeping more than other kids (β = 0.40, *p* = 0.001). T2 maternal-reports of sleeping less than other kids, however, were also related uniquely and negatively with T2 maternal-reports of talking/walking in sleep. Finally, T2 maternal-reports of trouble sleeping were associated uniquely with maternal-reports of being overtired (β = 0.43, *p* = 0.001) and sleeping less than other kids (β = 0.33, *p* = 0.006).

### 3.3. Exploratory Analyses

#### 3.3.1. Evaluation of Sex Differences

We next explored, without any a priori predictions, whether sex moderated the proposed associations with a multiple group analysis in which a fully unconstrained model (all paths and covariances freely estimated for both sexes) was compared to a fully constrained model (all regression paths and covariances set equal for both sexes). A significant χ^2^ difference test between the constrained and free-to-vary models indicated no differences across sex, Δχ^2^ (37) = 46.406, *p* = 0.13.

#### 3.3.2. Youth Self-Reports of Sleep Difficulties

We then evaluated the fit of an exploratory model identical to the primary model with one notable difference: T2 sleep variables were reported by the *adolescent*. Adolescent self-reported sleep data were not available at T1, and so we controlled for T1 maternal-reports in these models. There was one other difference: the YSR does not ask youth about the extent to which they are talking/walking in their sleep, and so this model included five (as opposed to six) T1 and T2 sleep variables. Table 2 shows the zero-order correlations between the study variables included in this model.

There was adequate fit to the data for this model: χ^2^ (20) = 40.77, *p* = 0.004, RMSEA = 0.05, 90% CI [0.028, 0.074], SRMR = 0.047, CFI =0.85, and no post hoc model fitting was performed. Of note, in this model, maternal-reports of nightmares at T1 predictively positively self-reports of nightmares at T2 (β = 0.19, *p* = 0.03). Similar prospective effects were found for T1 maternal-reports of trouble sleeping and T2 self-reports of trouble sleeping (β = 0.18, *p* = 0.02). Surprisingly, T1 maternal-reports of sleeping more than other kids were related *negatively* to T2 self-reports of sleeping more than other kids (β = −0.12, *p* = 0.04).

In terms of main effects, T1 exclusion/victimization was related positively to T2 self-reports of nightmares (β = 0.26, *p* = 0.02). Moreover, the interaction between T1 anxious-withdrawal and T1 exclusion/victimization significantly predicted T2 trouble sleeping (β = −0.19, *p* = 0.02). Simple slope analyses revealed that T1 anxious-withdrawal was a *negative* predictor of T2 trouble sleeping at high levels of peer exclusion/victimization (β = −0.18, *p* = 0.051) but was not a significant predictor at low levels of peer exclusion/victimization (β = 0.045, *p* = 0.36).

## 4. Discussion

Anxious-withdrawal during childhood and adolescence has been associated concurrently and prospectively with a host of psychological difficulties, including anxiety, depressive symptoms, and loneliness [1]. There is also evidence that anxiously-withdrawn behaviors interfere with positive peer interactions and relationships and instead promote such negative peer difficulties as peer exclusion and peer victimization [31]. However, researchers have yet to investigate whether anxiously-withdrawn behavioral tendencies also place youth at risk for physical health difficulties. The present study aimed to extend previous research by examining, for the first time, whether anxious-withdrawal during early adolescence is related concurrently and prospectively with six different types of sleep problems (nightmares, talking/walking in sleep, sleeping more than other kids, sleeping less than other kids, being overtired, and general trouble sleeping), as well as whether peer difficulties (in the form of peer victimization and exclusion) moderate these associations.

Consistent with expectations (Hypothesis #1), peer-nominated anxious-withdrawal was associated concurrently with several types of maternal-reported sleep problems, including nightmares and sleeping too much, in the zero-order correlational analyses. In path models, anxious-withdrawal was also related uniquely to T1 maternal-reports of sleeping more than other kids and trouble sleeping. Taken together, these findings suggest, for the first time, that adolescents who regularly withdraw from their peers due to social fears and anxieties during the daytime hours also experience difficulties in the nighttime. Although these findings will require replication, they may point to the need for a “24-h approach” in future research to fully understand the multitude of health difficulties associated with anxious-withdrawal. Such approaches, which track social interaction and activities continuously for 24 h, are becoming increasingly common in other areas of developmental and clinical psychology research (e.g., obesity research [42], personality disorder research [43]) but have not yet been utilized in studies of anxious-withdrawal. In fact, most studies of anxious-withdrawal not only neglect functioning and well-being during the nighttime hours but also neglect how anxiously-withdrawn youth fare when they are awake and *not* in school (or before and after school hours).

Young adolescents who are anxiously-withdrawn may experience sleep difficulties due to their nervous and negatively-biased social information processing and cognition tendencies (e.g., rejection-sensitivity, internal blame attributions [44]). Similar to those who are anxious as well as lonely and socially isolated, these negative cognitive styles may interfere with several aspects of the sleep process, such as falling asleep at the start of bedtime. Significantly, the findings from this study suggest that anxious-withdrawal may be related to some, but not all, types of sleep difficulties. As one example, anxious-withdrawal at T1 was related uniquely to T1 sleeping more than other kids and having trouble sleeping; anxious-withdrawal was not associated with T1 reports of sleeping less than other kids and talking/walking in sleep. This may point to the possibility that anxiously-withdrawn young adolescents struggle most with *initially* falling asleep and not *staying* asleep. Additional work with more nuanced assessments of sleep (e.g., physiological assessments of sleep), however, is needed to evaluate this hypothesis.

As shown in previous research, peer-nominated peer victimization and exclusion in this study was related concurrently to several types of sleep problems, including being overtired. Novel to this research, however, was the prospective and interactive effect of anxious-withdrawal and these peer problems on reports of trouble sleeping. Specifically, and consistent with Hypothesis #2, the results showed that anxious-withdrawal at T1 predicted increased maternal-reports of trouble sleeping at T2 for young adolescents who were highly excluded and victimized by their peers at school. The longitudinal relation between anxious-withdrawal and trouble sleeping was not significant for young adolescents who were low in peer victimization and exclusion. It is possible that these findings reflect increased worry, anxieties, and negative social cognitions experienced by anxiously-withdrawn young adolescents who also struggle in their relationships with their peers [45]. It seems that such cognitions and affect may, in turn, interfere with sleep. Past research has shown that youth with the intrapersonal risk factor of anxious-withdrawal suffer the most psychologically when they also struggle interpersonally with their peers [2,3,45]. The present findings, however, are the first to show similar intra- and interpersonal interactive effects as they pertain to a *physical* health outcome. The findings of the present study may suggest that anxiously-withdrawn young adolescents might benefit from explicit instruction on sleep hygiene. Peer problems can be difficult to change, especially without school-wide intervention efforts, but there is growing evidence to support the efficacy of cognitive–behavioral sleep interventions for not only adults but also adolescents [46,47,48]. Such therapies might not solve anxiously-withdrawn adolescents’ psychological and peer difficulties, but they might help them to be better rested when dealing with them.

Several limitations of this study should be acknowledged. First, even with longitudinal analyses, the present study does not provide evidence about causality. In addition, the conceptualization and analyses for our study were informed by other areas of research which assume that individual risk factors (e.g., anxiety) lead to the development of sleep difficulties over time [49,50]. However, it is certainly plausible that sleep difficulties, and the daytime exhaustion related to many of them, might lead some youth to not have the energy and positive affect necessary to engage with peers. Such direction of effects could not be examined in this study but should be evaluated in future research.

Second, the present study focused on peer victimization and exclusion, the two most commonly considered types of peer difficulties in research on anxious-withdrawal [31]. Although oftentimes discussed as conclusive, the evidence linking anxious-withdrawal and peer rejection (or active dislike) is actually quite mixed, especially in the few studies published on anxious-withdrawal and peer rejection with *adolescent* samples [31]. Nevertheless, future research on anxious-withdrawal and sleep should explore the potential contributions of peer rejection and other types of peer difficulties, including those involving friends (e.g., friendship instability, dissolution, and betrayal), which may also increase anxiously-withdrawn young adolescents’ distress and interfere with their sleep. In such research, measures of anxiety and negative cognitions should also be included to evaluate the proposed mechanisms of influence in this study [50,51]. The lack of such assessments in this study is another significant limitation.

Third, this study was further limited by its reliance on *maternal*-reports of sleep difficulties. We did explore self-reports of sleep difficulties at T2, but we are cautious to interpret such findings given the lack of T1 self-reports of sleep difficulties. That said, the results did differ somewhat from those with the maternal-reports of sleep difficulties, which we think may point to the importance of multiple informants in future research in this area of research [52,53]. Perhaps multiple informant assessments might help make sense of several unexpected findings found in this study, such as why T1 exclusion/victimization predicted *decreases* in maternal-reports of trouble sleeping over time and T1 anxious-withdrawal predicted *decreases* in maternal-reports of talking/walking in sleep from T1 to T2; these effects were also not found when T2 self-reports of sleep difficulties were considered.

## 5. Conclusions

Despite these limitations, the present study extends past research with its consideration of anxious-withdrawal in relation to physical health difficulty. The findings linking anxious-withdrawal concurrently and prospectively to sleep difficulties are novel and should set the stage for future research on anxious-withdrawal that conceptualizes adjustment difficulties more broadly to include physical health difficulties. Also noteworthy was the interaction effect between anxious-withdrawal and peer exclusion/victimization in the prediction of trouble sleeping. This result is the first to show that anxiously-withdrawn young adolescents’ peer difficulties contribute to physical heath difficulties (just as they contribute to their social and psychological health difficulties). Thus, we hope this latter finding helps to further highlight the importance of considering peer difficulties in the study of anxious-withdrawal, and perhaps after replication, informs intervention and prevention efforts with youth who regularly withdraw from their peers due to social fears and anxieties.

## Figures and Tables

**Table 1:** Zero-order Correlations and Descriptive Statistics.

	1	2	3	4	5	6	7	8	9	10	11	12	13	14
1. Anxious-withdrawal T1														
2. Exclusion/Victimization T1	0.320 **													
3. Nightmares T1	0.122 *	0.127 *												
4. Overtired T1	0.101	0.071	0.119 *											
5. Sleeps less T1	0.039	0.037	0.112 *	0.262 **										
6. Sleeps more T1	0.175 **	0.055	0.134 **	0.104 *	0.013									
7. Talks/walks T1	−0.052	−0.054	0.173 **	0.034	−0.005	0.082								
8. Trouble sleeping T1	0.198 **	0.175 **	0.288 **	0.210 **	0.301 **	0.08	0.181 **							
9. Nightmares T2	0.089	0.068	0.244 **	0.057	−0.059	0.004	0.186 *	0.053						
10. Overtired T2	0.1	0.275 **	0.232 **	0.300 **	0.15	0.097	0.021	0.212 **	0.261 **					
11. Sleeps less T2	0.008	0.008	0.015	0.007	0.436 **	0.026	−0.074	0.128	0.036	0.187 *				
12. Sleeps more T2	0.194 *	0.081	0.023	−0.002	0.048	0.202 **	0.018	0.245 **	0.066	0.388 **	0.272 **			
13. Talks/walks T2	−0.145	−0.115	0.011	−0.006	−0.055	0.008	0.433 **	−0.028	0.297 **	0.054	−0.142	0.071		
14. Trouble sleeping T2	0.225 *	0.247 **	0.178 *	0.208 **	0.222 **	0.15	0.074	0.374 **	0.311 **	0.451 **	0.307 **	0.163 *	−0.019	
*M*	−0.016	−0.005	0.168	0.223	0.168	0.097	0.112	0.125	0.138	0.204	0.174	0.078	0.133	0.156
*SD*	0.837	0.605	0.381	0.463	0.425	0.344	0.34	0.394	0.379	0.446	0.439	0.29	0.374	0.38

*Note.* Sleep variables were mother-reported, anxious-withdrawal and victimization/exclusion were based on peer nominations; T1 = Time 1 or Grade 5/6; T2 = Time 2 or Grade 8; * *p* < 0.05; ** *p* < 0.001.

**Table 2:** Zero-order Correlations and Descriptive Statistics for Maternal-reported (T1) and Self-reported (T2) Sleep Difficulties.

	1	2	3	4	5	6	7	8	9	10	11	12
1. Anxious-withdrawal T1												
2. Exclusion/Victimization T1	0.320 **											
3. Nightmares T1	0.122 *	0.127 *										
4. Overtired T1	0.101	0.071	0.119 *									
5. Sleeps less T1	0.039	0.037	0.112 *	0.262 *								
6. Sleeps more T1	0.175 **	0.055	0.134 **	0.104 *	0.013							
7. Trouble sleeping T1	0.198 **	0.175 **	0.288 **	0.210 **	0.301 **		0.08					
8. Nightmares T2	0.019	0.166	0.167 *	0.043	0.026	−0.023	−0.042					
9. Overtired T2	0.009	0.088	−0.006	0.067	0.095	0.197 **	0.065	0.231 **				
10. Sleeps less T2	−0.106	−0.09	−0.088	0.027	0.162 *	−0.047	−0.007	0.092	0.206 **			
11. Sleeps more T2	−0.042	0.017	−0.122	−0.076	−0.114	0.008	−0.067	−0.019	0.065	−0.053		
12. Trouble sleeping T2	−0.08	−0.084	0.022	0.038	0.211 **	0.089	0.116	0.171 *	0.429 **	0.408 **	−0.089	
*M*	−0.016	−0.005	0.168	0.223	0.168	0.097	0.125	0.477	0.465	0.465	0.269	0.374
*SD*	0.837	0.605	0.381	0.463	0.425	0.344	0.394	0.577	0.652	0.643	0.562	0.614

*Note.* All T1 sleep variables were maternal-reported, and all T2 sleep variables were self-reported, and T1 anxious-withdrawal and victimization/exclusion were based on peer-nominations; T1 = Time 1 or Grade 5/6; T2 = Time 2 or Grade 8; * *p* < 0.05; ** *p* < 0.001.

## Data Availability

De-identified data and code will be made available upon reasonable request to the first author.
